# Timothy Syndrome and CACNA1C-Related Disorder: First International Language and Management Guidelines Consensus Statement

**DOI:** 10.21203/rs.3.rs-8058536/v1

**Published:** 2025-11-17

**Authors:** Jack F. G. Underwood, Katherine W. Timothy, Holly Tyroll, Rebecca J. Levy, Ivy E. Dick, Geoffrey S. Pitt, Elizabeth M. Tunbridge, Anwar Baban, Gemma Wilkinson, Nicola A. L. Hall, Georgia Sarquella Brugada, Rosemary Bauer, Dominic Abrams, Jeremy Hall, Timothy Syndrome Foundation, Timothy Syndrome Alliance (TSA)

**Affiliations:** Cardiff University; The Timothy Syndrome Foundation; Cardiff University; Stanford University; University of Maryland, Baltimore; Cornell University; University of Oxford; ERN GUARD-Heart; Cardiff University; University of Oxford; ERN GUARD-Heart; The Timothy Syndrome Foundation; Boston Children’s Hospital; Cardiff University; The Timothy Syndrome Foundation; Timothy Syndrome Alliance (TSA)

**Keywords:** Timothy Syndrome, CACNA1C-Related Disorders, Long QT, cardiac arrhythmia, syndactyly, developmental delay

## Abstract

Timothy Syndrome is a multisystemic genetic disorder, classically characterised by prolonged QT interval and subsequent cardiac arrhythmias, neurodevelopmental disorders including developmental delay and autism, and syndactyly or hip dysplasia. It is caused by variants in the *CACNA1C* gene, which encodes the widely expressed Ca_v_1.2 voltage-gated calcium channel. Since it’s characterisation in 2004, the spread of variants in *CACNA1C* associated with Timothy Syndrome has expanded. With advances in sequencing and the inclusion of *CACNA1C* in genomic screening, further variants have been identified presenting with incomplete features of Timothy Syndrome or further aligned phenotypes which are inconsistent with the original description. In the absence of a formal nomenclature, these presentations have been reported in a proliferation of ill-defined terms, e.g. Atypical Timothy Syndrome. At the same time, advances in knowledge and therapeutics have improved morbidity and life expectancy for these individuals when appropriately identified and managed. Here, we present guidelines for the diagnosis of individuals presenting with variants in *CACNA1C*, developed by an international panel of experts through Delphi consensus with the involvement of the *CACNA1C* community. We formalise the language around syndromic presentations linked to *CACNA1C* variants, reassert and demarcate the classical Timothy Syndrome phenotype, and define a new syndrome, CACNA1C-Related Disorder. Finally, we present minimum expected standards of clinical care for individuals with CACNA1C-Related Disorder or Timothy Syndrome, with implications for long-term management and improved outcomes for affected individuals.

## Background

Timothy Syndrome (TS) (Online Mendelian Inheritance in Man (OMIM) entry #601005) is a multisystemic disorder incorporating physical, neurodevelopmental and psychiatric features. It was first described in 1992, through cases of a novel prolonged QT arrhythmia syndrome associated with syndactyly (finger and toe webbing) (1, 2). It was formally characterised in 2004 through the work of the Keating and Splawski labs (3), and named after Katherine Wilson Timothy (KWT), Clinical Coordinator and key force behind the search and triangulation of the genetic locus of TS.

The causative locus for TS was isolated to a missense p.G406R variant in the *CACNA1C* gene at 12p13.33, encoding the Ca_V_1.2 L-type voltage gated calcium channel (3). Its ultra-rare presentation was coupled with significant early-life mortality, which has been reduced with the uptake of screening for prolonged QT in infants born with syndactyly. Population prevalence estimates remain unknown, though some affected individuals are now living well into adulthood with proactive healthcare management and monitoring. KWT’s 2024 Natural History Study of TS included 87 cases (4), whilst the Timothy Syndrome Alliance’s (TSA’s) global *CACNA1C* Community Registry includes 104 individuals as of October 2025. Through efforts by KWT, the Timothy Syndrome Foundation (TSF) and the Timothy Syndrome Alliance (TSA) to raise awareness, the number of identified living individuals with *CACNA1C*-Related Disorders has risen in the past five years alone from ~ 40 to > 200, with rapid growth coinciding with the inclusion of *CACNA1C* in a range of gene-screening panels and clinical implementation of comprehensive non-targeted next-generation (exome or genome) sequencing.

Since the first description of Timothy Syndrome, the phenotype has expanded, as further loci and affected organ systems have been identified. An early advance was the recognition that the thirteen children in whom TS was first identified all had the identical p.G406R variant in exon 8A (3). Exons 8 and 8A can be alternatively spliced in a mutually exclusive manner, and in 2005 a further individual with TS features, most notably an arrhythmia syndrome associated with marked QT prolongation but no syndactyly, was identified (4, 5). This individual, with a p.G406R variant present in exon 8 and congenital hip dysplasia, was the first of a small number that led to the definition of TS Type II (TS2) linked to p.G406R in exon 8, differentiated from TS Type I (TS1) in exon 8A. An additional case in the same study, of an individual with a p.G402S variant in exon 8 and with a similar phenotype, was also included in TS2, extending the molecular diagnostic criteria to other loci within *CACNA1C* for the first time (5). It should be noted that the exon 8/8A nomenclature is not consistent in the literature, with many early papers referring imprecisely to Timothy Syndrome across exons 8 and 8A. Here, we utilise the nomenclature originally described in Splawski *et al*, with TS1 comprising a phenotype including syndactyly, and TS2 featuring hip dysplasia (5).

The initial descriptions of TS invariably included prolongation of the QT interval, a feature which was soon classified as Long QT syndrome type 8 (LQT8). However, non-cardiac phenotypes are more variable. Thus, subsequent work focused on individuals with isolated prolonged QT intervals without the broader range of TS features (6). Initial studies indicated that these individuals did not exhibit cognitive impairment, facial dysmorphology or other non-cardiac features (6, 7). LQT8 was observed with pathogenic variants at loci on the gene distinct from TS, for example, p.P857R in Boczek *et al*’s study of 15 members of a multi-generational family (6), and p.R858H in Gardner *et al*’s study of 26 individuals from a family across five generations (7). Systematic screening of individuals in long QT databases yielded further case series and novel loci amongst cohorts internationally (8, 9). Such studies predominantly focused on cardiac manifestations, and reporting of extra-cardiac features was further complicated by cardiac arrest or hypoxic brain injuries causing neurological injury secondary to the primary arrhythmias. Moreover, most of these early studies did not include long-term follow-up evaluation, complicating the assessment of a cardiac-specific phenotype.

Some reported cases of novel pathogenic *CACNA1C* rare variants have included symptoms that mirror or physiologically-oppose TS or LQT8, such as short QT duration or Brugada ECG patterns. Short QT syndrome (SQTS) and Brugada Syndrome (BrS) were first reported in individuals with *CACNA1C* p.A39V and p.G490R variants in 2007, not long after the definition of TS, however the evidence for such variants as a genetic aetiology of SQTS and BrS is disputed (10–12). As with LQT8, subsequent case series and reports drawn from clinical cohorts of individuals with established BrS or early repolarisation syndromes have proposed a spread of loci across the *CACNA1C* gene linked to a multisystemic phenotype (13–15). These studies, however, are predominantly single cases, lack familial genetic testing pedigrees, and often variants presented occur at relatively common population frequencies. In 2018 the Clinical Genome Resource (ClinGen) re-examined the gene-disease relationship for *CACNA1C* and Brugada Syndrome, and classified the evidence supporting a causal role for *CACNA1C* in Brugada Syndrome as limited (12). Subsequent international meetings held on topics of ultra-rare cardiac diseases have supported this opinion, based on insufficient case-level data and lack of functional validation evidencing an association between *CACNA1C* and Brugada Syndrome.

**Figure 1:**
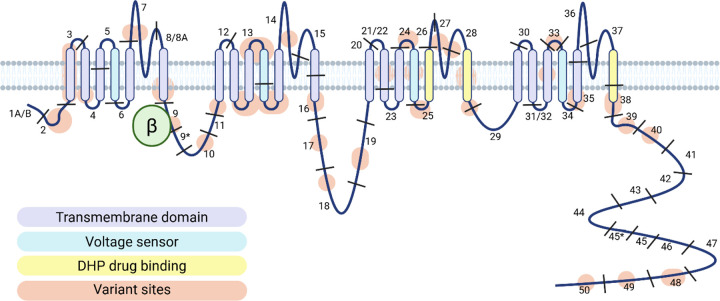
Published variants in CACNA1C mapped to a model of the Ca_v_1.2 protein Based upon review of published variants undertaken to September 2025. See Supplementary Material for list of variants and publications. Created in BioRender. Tunbridge, E. (2026) https://BioRender.com/b078y2i

Beyond cardiac presentations, rare variants in the *CACNA1C* gene have also been implicated as the cause of syndromic presentations in sporadic case series and reports (Fig. 1)(16–20). These symptomatically overlap with the original phenotype ascribed to TS, but include some, but not all, features. Neurodevelopmental disorders are frequently observed: developmental delay, intellectual disability, epilepsy, autism, attention-deficit hyperactivity disorder (ADHD), and hypotonia (16, 17, 19, 20). Efforts establishing this phenotype over the past five years has led it to be added to multiple reference sources, including OMIM (#620029), Orphanet, Wikipedia and Gene Reviews (21). The largest and most rigorous case series and literature reviews now confirm that rare variants in the *CACNA1C* gene cause a highly penetrant multisystemic disorder, but no single review examines all potentially implicated phenotypic features (Table 1).

As the literature on rare pathogenic variants in *CACNA1C* has expanded, the language and nomenclature used to describe those presentations has become chaotic and confusing. Beyond TS and LQT8, a range of other terms can be found across published literature and public-facing media, including Atypical Timothy Syndrome, Timothy Syndrome Type 3, Timothy Syndrome Variant, Timothy Syndrome-like *CACNA1C* Disorder, Cardiac-only Timothy Syndrome, *CACNA1C*-Related Disorders, and *CACNA1C*-Associated Neurological Disorders. These terms have no clear, established or defined clinical meaning. The number of identified individuals with rare pathogenic *CACNA1C* variants is increasing, support groups and communities are growing, and therapeutic treatments are in development (22). There is therefore a need for clarity to guide families, researchers and clinicians. In this work, we set out to achieve cohesion through convening an international network of experts and engaging with the leading TS charities. This resulting consensus document outlines a standard language definition for individuals presenting with variants suspected to be pathogenic in *CACNA1C*, along with the first guidelines for their diagnosis and management.

## Methods

Development of the *CACNA1C* consensus guideline was modelled on the Delphi Process (26), incorporating multiple rounds of expert discussion, followed by engagement and co-production with the TS and *CACNA1C* community. The *CACNA1C* language consensus group consisted of twelve participants from ten institutions across the USA, UK and Europe, comprising clinicians, clinical scientists and researchers. Discussions occurred via video conference calls, e-mail communications and draft file outcomes exchanges.

**Figure 2:**
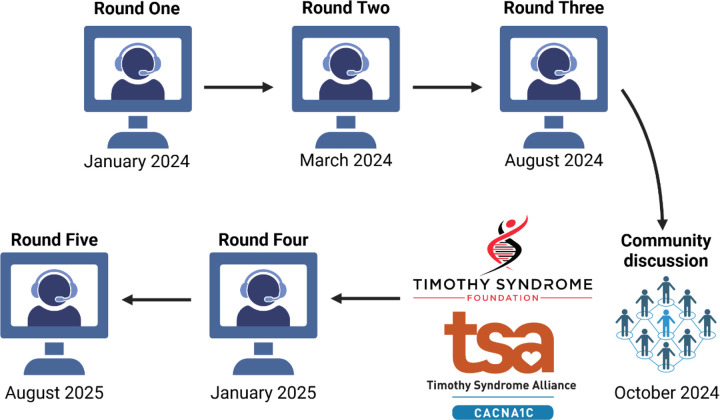
Timeline of meetings and public engagement of the CACNA1C language consensus group This figure was created in BioRender. Underwood, J. (2026) https://BioRender.com/etvxcc3

Key issues for discussion were initially identified following the *Connect CACNA1C Global Network Conference* held by the Timothy Syndrome Alliance (TSA) in June 2023. The language consensus group was convened, and after three rounds of discussions from January to August 2024 (Fig. 2), language options were presented to the TS and *CACNA1C* community for input, along with identification of any further relevant issues. Individuals with *CACNA1C* variants and their caregivers were contacted through social media, support groups and the Timothy Syndrome Alliance (TSA) community mailing lists, reaching > 200 families. The community voted on language options, which were then discussed along with further outcomes to generate consensus recommendations at a further two working group meeting rounds.

### Clinical Diagnostic Criteria

#### Clinical features of Timothy Syndrome

As noted, TS Type 1 was defined through cardinal features of prolonged QT interval, cardiac arrhythmia and syndactyly in individuals with a p.G406R gene variant in exon 8A of *CACNA1C* (3). Further features included autism, developmental delay, seizures, baldness at birth, characteristic facies (flattened nasal bridge, low-set ears, small upper jaw, thin upper lip), small teeth, hypoglycaemia, hypothermia, and hypotonia (3, 23). Inclusion of individuals with p.G406R variants in exon 8 associated with a similar phenotype but featuring hip dysplasia led to the development of TS Type 2 (5). These remain the classical signs of TS Type 1 (hereafter TS1) and Type 2 (TS2), and our consensus reaffirmed the importance of these diagnostic entities. The p.G406R gene variant is highly penetrant, and we therefore expect individuals with p.G406R variants to present with symptoms of TS1 or TS2, dependent on whether the p.G406R variant is present in exon 8 or 8A (21).

**Figure 3:**
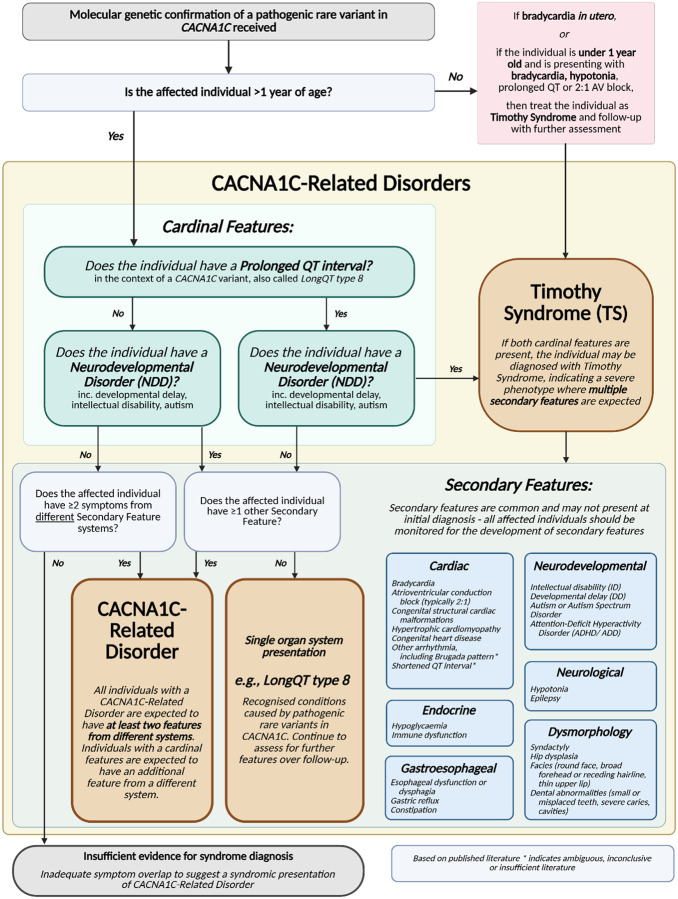
Flow chart for syndromic interpretation and diagnosis in individuals with pathogenic CACNA1C rare variants This figure was created in BioRender. Underwood, J. (2026) https://BioRender.com/jlnh24q

In Splawski *et al*’s work defining TS2, they extended the loci to include p.G402S variants, although this appears to have more variable penetrance (5). Further case reports and series have presented individuals with the classical phenotypic features of TS, but novel *de novo* gene variants, notably p.R324W (16), p.V403M (16), p.E407G (27), p.E407A (28), p.C1021R (29, 30), p.I1166T (6, 9, 28), and p.A1473G (31). On this basis, TS1 and TS2 diagnoses should be assigned based on phenotypic syndromic features and not be dependent upon the specific p.G406R loci. Any individual with a rare single nucleotide variant within the *CACNA1C* gene presenting with the classic dyad of prolonged QT interval and a neurodevelopmental disorder (e.g. developmental delay, intellectual disability, autism) should be diagnosed as TS following appropriate clinical genetic medical evaluation. In Fig. 3 below, we outline a flow chart for diagnostic interpretation of *CACNA1C* pathogenic rare variants based upon assessment and examination of all potential syndromic features.

These individuals should be further assessed across all potentially affected organ systems, with ongoing follow-up on an annual basis to monitor for further symptoms driven by developmental changes. Of note, individuals with pathogenic variants in *CACNA1C* may present solely with bradycardia whilst *in utero*, and in the first year of life with bradycardia or hypotonia, prior to the development of other features. Neonates or infants may show prolonged QT intervals on electrocardiography, with or without 2:1 functional atrioventricular block (secondary to the prolonged QT). All individuals identified *in utero* or in the first year of life displaying these features should be treated as suspected TS and followed up closely with proactive intervention and management.

#### CACNA1C-Related Disorders

**Figure 4:**
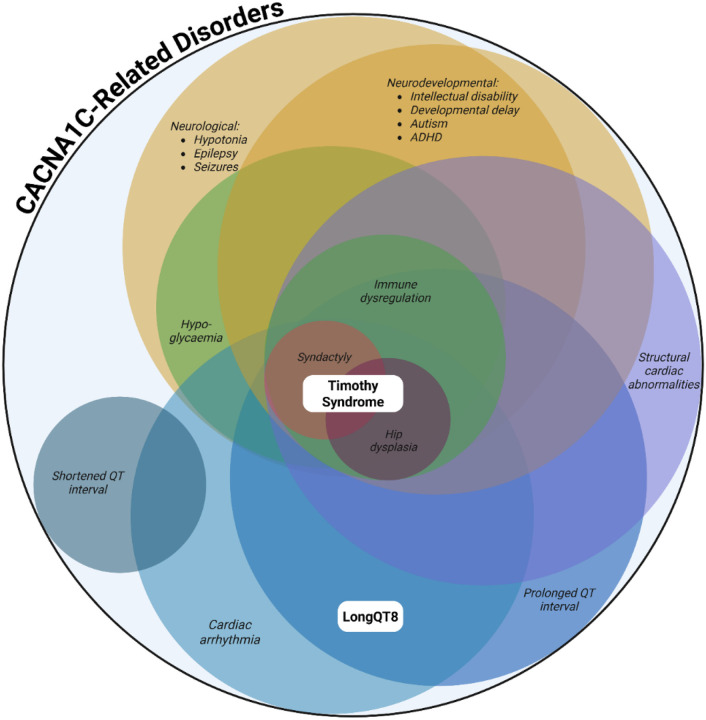
Euler diagram of diagnoses and syndromes featuring CACNA1C gene changes This figure was created in BioRender. Underwood, J. (2026) https://BioRender.com/335c7zp

Individuals with other, non-G406R variants in the *CACNA1C* gene may also present with some, but not all, features of TS (16, 17, 19, 20, 32). Potential diagnostic and nomenclature structures were distilled to two options through the language consensus working group meetings, which were presented to the *CACNA1C* community. 31 individuals with *CACNA1C* variants or their families responded to requests for input, voting by 26 to 3 for the adoption of a new CACNA1C-Related Disorders diagnostic term (with two individuals suggesting further options). This new definition provides an umbrella term for all individuals with pathogenic rare variants in *CACNA1C* beyond those with TS1 or TS2 phenotypes, as laid out in Figs. 3 and 4. This serves to bring those with LQT8 into the wider community, and to include those individuals who previously did not have a formal diagnosis, allowing them improved access to medical care and therapies. Within the CACNA1C-Related Disorders (CRDs) umbrella, syndromic presentations of pathogenic *CACNA1C* rare variant with multiple organ system involvement fitting published literature but inconsistent with TS can be diagnosed with CACNA1C-Related Disorder (Fig. 3). CACNA1C-Related Disorder (CRD) aligns the *CACNA1C* gene with the movement towards gene-based nomenclature, which has been observed across the field and particularly in channelopathies, e.g., CACNA1A-related disease (33), SCN2A-related disorders (34), and SCN8A-related disorders (35).

##### Cardiac presentations of CACNA1C-Related Disorders

Individuals may initially present with isolated cardiac features, encompassing a range of phenotypes featuring isolated prolonged QT interval, arrhythmias, hypertrophic cardiomyopathy, congenital heart disease, sinus node disease and congenital structural cardiac abnormalities (Fig. 3). The symptomatic presentation of LQT8 in individuals with rare *CACNA1C* variants has been robustly demonstrated (4, 18, 36, 37). Further cardiac phenotypes have previously been linked with *CACNA1C* rare variants, including Brugada Syndrome (BrS) (14, 38, 39), short QT syndrome (SQTS) (10, 14, 15), and hypertrophic cardiomyopathy, congenital heart disease and sinus node disease (6, 40, 41), overlapping and characterised as cardiac-only TS (36, 37, 42). For BrS and SQTS studies provide only limited support for a link between *CACNA1C* and those phenotypes, and the causal relationship is disputed (11), as further evidenced by ClinGen’s re-evaluation of the literature (12). We recognise that there is no consensus on this evidence, with a need for further in-depth assessments and functional analyses of posited loci.

Many existing case reports on individuals with LQT8 are limited to only cardiovascular symptom profiling; therefore, concern was raised throughout the consensus group meetings that this may represent under-reporting or under-assessment of non-cardiac phenotypic features. These single organ-system presentations may be due to splice variation, mosaicism, or decreased penetrance, but all individuals presenting in such a manner require a full, holistic, multisystemic medical work-up. Individuals presenting with single organ system presentations of a pathogenic *CACNA1C* rare variant (Fig. 3), for example LQT8, fall within the CACNA1C-Related Disorders umbrella term. Individuals identified via single organ-system presentations early in childhood often present later in development with further symptoms as *CACNA1C* expression regulation changes (4), and therefore, ongoing observation and follow-up is advised. In those with *CACNA1C* variants, each individual’s QT intervals can be highly variable with wide ranges, and cardiac risk does not clearly correlate with observed QT duration (43). Furthermore, some patients may initially present with borderline QT prolongation, which may evolve to LQT8 or place them at risk for acquired long QT syndromes. This evolution of symptoms further supports the umbrella term of CACNA1C-Related Disorders for all patients presenting with a pathogenic *CACNA1C* variant, and the need for regular cardiac monitoring as a baseline standard of care.

### Molecular Diagnostic Criteria

#### Functional interpretation

The initial novel missense p.G406R variant first identified in exon 8A results in a gain-of-function, manifesting as significant changes in channel activation, voltage-dependent inactivation (VDI) and calcium-dependent inactivation (CDI)(3, 5, 44, 45). Electrophysiological studies have demonstrated that this combination of effects is not isolated to the p.G406R variant, but the TS phenotype appears associated pathophysiologically with specific changes in the electrophysiological properties and functions of the Ca_v_1.2 channel. These include hyperpolarised left-shifts of activation curves coupled to altered CDI and VDI, with notable differences observable within this grouping between individual variants such as p.G406R and p.G402S (23, 44, 45). Subsequent work has shown that these variants have further effects, including decreased synaptic inhibition, defects in cortical differentiation, altered long-term potentiation, and altered cortical interneuron migration (46–48). Variant function can therefore be understood to be more complex than simple ‘gain- or loss-of-function’, and many variants result in changes with mixed effects, as recently reviewed by Bauer *et al* (6, 9, 18, 32, 45).

Further complicating this picture is evidence of both non-linear relationships between *CACNA1C* variants and Ca_V_1.2 expression profiles (44). Variants may show increased cell surface expression due to decreased degradation (6, 8), while some variants (notably p.G406R) increase basal transcription through excitation-transcription coupling (49), or alter neuronal gene expression (50), with enhanced activation of the Ca_v_1.2 channel driving multiple pathological mechanisms at both the cellular and network level (44, 51, 52). Expression of Ca_v_1.2 and development of symptoms at a system or organ level are subsequently dependent on transcription of the pathological variant. Ca_v_1.2 is widely expressed in almost all tissues and is critical for development (3, 4, 53). Evidence from long-read sequencing suggests that *CACNA1C* incorporates at least 47 constituent exons, with > 240 novel transcripts, and those transcripts demonstrate developmental stage and tissue-specific splicing, which are predicted to alter channel function (54). These mechanisms, in combination with germline mosaicism, are hypothesised to drive the irregular penetrance and phenotypic variability seen across CRDs (4, 18, 19, 23, 29).

Due to this complexity, clinical pathogenicity should not be extrapolated based upon inferred gain- or loss-of-function. We recommend supporting clinical variant interpretation with functional testing of the variant in established model systems. Tools and literature supporting variant interpretation are predicted to evolve, and therefore we advise periodic re-testing and re-evaluation utilising updated evidence. This guidance supports the recommendation that diagnoses should be made on a syndromic basis, and pathogenicity cannot yet be inferred from gain- or loss-of-function descriptors alone.

#### Guidelines for Clinical Care

Due to the widespread but variable expression and mixed effects of *CACNA1C* gene variants, presentations are individual specific but frequently multisystemic. All individuals with a *CACNA1C* pathogenic or likely pathogenic variant require a full multidisciplinary team clinical work-up, to include, at a minimum cardiac, neurodevelopmental, musculoskeletal and endocrine reviews. We expect individuals to show an evolution of risk and development of new syndromic features over time, secondary to changes in expression of *CACNA1C* and the Ca_v_1.2 channel over the lifespan, necessitating consistent follow-up. For individuals with variants of unknown or uncertain significance (VUS) in the *CACNA1C* gene, it is reasonable to clinically screen for other organ involvement to determine if there is additional clinical evidence of a CRD.

Cardiac features of CRDs including TS, as the most reported and historically most lethal phenotypic presentation, have received significant therapeutic study (4, 18). Recent reviews and case series have demonstrated that beta-blocker medication and implantable cardioverter-defibrillators (ICDs) are the most effective current treatments (23, 36, 55). Surgical interventions, through left cardiac sympathetic denervation to attenuate heterogeneous sympathetic myocardial innervation, have also demonstrated efficacy (4, 56). Calcium-channel blockers, perhaps counterintuitively, are not effective in the management of any CRDs (including TS) due to the complex gating changes in Ca_v_1.2 (4, 45, 57). Implantation of an ICD early in life decreases mortality risk, and this change in practice has led to a dramatic increase in life expectancy of TS children since the syndrome was first described (4, 23, 57).

No other comprehensive organ-level systematic reviews have been undertaken in TS or more broadly across CRDs. Timothy *et al*’s 2024 Natural History Study of TS covers a broad range of phenotypic features in depth, including discussion of key areas for clinical concern (4). As TS and CRD children survive longer, there is evidence of emerging mortality risks from further syndromic features (4, 20, 21). Epilepsy is prevalent across CRDs, and seizures may be refractory to standard treatments (16, 17, 20, 32). Individuals with CRDs where there is suspicion of seizure activity should have neurologic evaluation and seizure rescue medication available in their home, work or school environments. Hypoglycaemia has been noted in CRDs (3–5), and recent work has demonstrated that this is through dysregulated glucose homeostasis, absent hyperinsulinism, and defects in glucagon secretion (58). Individuals with *CACNA1C* variants are at risk of hypoglycaemia, particularly when in states of physiological stress, including viral illnesses, which may compound with arrhythmias and lower seizure thresholds to increase the risk of sudden death. Those with CRDs (including TS) should therefore monitor blood glucose levels routinely and have rescue kits (e.g. glucose tablets or gel, fruit juice or sugary drinks/candy) available for use in episodes of low blood sugar.

Further features of CRDs will require long-term therapies and interventions and are treated symptomatically and individually. Responses to anaesthesia are reportedly abnormal, and care should be taken with all sedation (4). Many children with CRDs have frequent infections, likely due to altered immunological function (3, 4). Abnormal dentition, frequent cavities and small displaced teeth requiring surgical intervention have commonly been reported (3, 4, 19). Gastroesophageal reflux, frequent vomiting and congenital gastrointestinal defects have been reported across CRDs (4, 30). Chronic constipation is seen in > 80% of individuals with TS, and anecdotally stated to be a significant issue amongst CRDs (3, 4). The majority of children with CRDs will experience some developmental delay, particularly of speech (4, 19, 20). Autism is a highly penetrant phenotypic feature across CRDs, and other neurodevelopmental conditions (e.g. ADHD), neurosensory and neuromuscular features such as hypotonia complicate this presentation. All children with CRDs should be provided with individualised, tailored support in education and work environments.

Long-term evolution of features and outcomes, particularly in those without prolonged QT, are not known, although several individuals are now well into mid-life with a range of functional outcomes (4, 20). Based on the above guidance, all individuals with a *CACNA1C*-Related Disorder should have as a minimum standard of care:

- Annual cardiac and endocrine review including echocardiography (ECG/EKG)
- Implantable cardioverter-defibrillators in those with prolonged QTc, and loop recorders in those with borderline QTc
- Annual neurodevelopmental reviews whilst < 18 years of age
- Periodic home ambulatory EEG to identify potential seizure activity
- Home glucose monitoring, hypoglycaemia and seizure rescue kits with appropriate training.

## Conclusions

In the thirty years since Keating *et al* first described a ‘heart-hand’ syndrome, and twenty years since this was formalised as Timothy Syndrome, the landscape of phenotypic features associated with rare pathogenic variants in the *CACNA1C* gene has changed substantially. TS remains the most severe presentation, but genomic loci linked to this syndromic phenotype have extended beyond the classical p.G406R variant. Meanwhile, a range of cases have been reported with overlapping or contrasting phenotypic features and novel rare *CACNA1C* variants. Here, through expert consensus and community engagement, we summarise updated phenotypes and features for TS and its associated presentations. We define *CACNA1C*-Related Disorders, a new umbrella phenotypic syndrome, capturing the range of classical and newly recognised presentations. Finally, we present the first guidelines on standards of diagnostic and clinical care for individuals presenting with a rare *CACNA1C* gene variant, driven by the evidence that with appropriate interventions, these individuals can live longer, fulfilling lives.

## Supplementary Material

Supplementary Files

This is a list of supplementary files associated with this preprint. Click to download.

- SupplementaryMaterial.pdf

## Figures and Tables

**Table 1:** Overview of case series and literature reviews examining Timothy Syndrome, CACNA1C-related disorder and long QT syndrome 8

	Timothy *et al,* A Natural History of Timothy Syndrome, 2024 (4)	Walsh *et al,* A multicentre study of patients with Timothy syndrome, 2017 (23)	Rodan *et al,* Phenotypic expansion of *CACNA1C*-associated disorders to include isolated neurological manifestations, 2021 (16)	Borbás *et al,* Geno- and phenotypic characteristics and clinical outcomes of *CACNA1C* gene mutation associated Timothy syndrome, “cardiac only” Timothy syndrome and isolated long QT syndrome 8: A systematic review, 2022 (24)	Levy *et al,* A cross-sectional study of the neuropsychiatric phenotype of *CACNA1C*-related disorder, 2022 (20)	Matthews *et al,* International Cohort of Neonatal Timothy Syndrome, 2024 (25)	Cipriano *et al,* Expanding the Phenotype of the *CACNA1C*-Associated Neurological Disorders in Children: Systematic Literature Review and Description of a Novel Mutation, 2024 (19)
*Sample size*	87	6	25	59	24	44	35
*Prolonged QT interval/arrhythmia*	Identified	Identified	Identified	Identified	Identified	Identified	Excluded ^
*Shortened QT interval*	Not discussed	Not discussed	Not observed	Excluded $	Not discussed	Not discussed	Excluded ^
*Intellectual disability*	Not discussed	Not discussed	Identified	Not discussed	Identified	Not discussed	Identified
*Developmental delay*	Identified	Identified	Identified	Identified	Identified	Not discussed	Identified
*Syndactyly*	Identified	Identified	Identified	Identified	Not discussed	Identified	Not discussed
*Hip dysplasia*	Identified	Identified	Identified	Not discussed	Not discussed	Not discussed	Not discussed
*Structural cardiac malformations*	Identified	Identified	Identified	Not discussed	Not discussed	Identified	Identified
*Arrhythmia*	Identified	Identified	Identified	Identified	Not discussed	Identified	Excluded ^
*Autism*	Identified	Not discussed	Identified	Identified	Identified	Not discussed	Identified
*ADHD*	Identified	Not discussed	Identified	Not discussed	Identified	Not discussed	Not discussed
*Hypoglycaemia*	Identified	Identified	Not discussed	Identified	Not discussed	Identified	Not discussed
*Hypotonia*	Identified	Not discussed	Identified	Not discussed	Identified	Not discussed	Identified
*Epilepsy/seizures*	Identified	Identified	Identified	Identified	Identified	Not discussed	Identified
*Immune dysfunction*	Identified	Not discussed	Not discussed	Identified	Not discussed	Not discussed	Not discussed

Identified = assessed and observed in cases in study; Not discussed = not assessed in cases in study;

$= Borbás et al excluded case reports on short QT syndrome and Brugada syndrome associated with CACNA1C from their review;

^= Cipriano et al excluded case reports with “documented cardiac conduction defects”
